# EfficientRMT-Net—An Efficient ResNet-50 and Vision Transformers Approach for Classifying Potato Plant Leaf Diseases

**DOI:** 10.3390/s23239516

**Published:** 2023-11-30

**Authors:** Kashif Shaheed, Imran Qureshi, Fakhar Abbas, Sohail Jabbar, Qaisar Abbas, Hafsa Ahmad, Muhammad Zaheer Sajid

**Affiliations:** 1 Department of Multimedia Systems, Faculty of Electronics, Telecommunication and Informatics, Gdansk University of Technology, 80-233 Gdansk, Poland; kashif.shaheed@pg.edu.pl; 2 College of Computer and Information Sciences, Imam Mohammad Ibn Saud Islamic University (IMSIU), Riyadh 11432, Saudi Arabia; sjjabar@imamu.edu.sa (S.J.); qaabbas@imamu.edu.sa (Q.A.); 3 Centre for Trusted Internet and Community, National University of Singapore (NUS), Singapore 117411, Singapore; fakhar.5@nus.edu.sg; 4 Department of Computer Software Engineering, Military College of Signals, National University of Science and Technology, Islamabad 44000, Pakistan; hahmad.mscs22mcs@student.nust.edu.pk (H.A.); msajid.msse-27mcs@student.nust.edu.pk (M.Z.S.)

**Keywords:** agriculture, classification, deep learning, transfer learning (TL), CNNs, ResNet-50, vision transformer (ViT), potato diseases

## Abstract

The primary objective of this study is to develop an advanced, automated system for the early detection and classification of leaf diseases in potato plants, which are among the most cultivated vegetable crops worldwide. These diseases, notably early and late blight caused by *Alternaria solani* and *Phytophthora infestans*, significantly impact the quantity and quality of global potato production. We hypothesize that the integration of Vision Transformer (ViT) and ResNet-50 architectures in a new model, named EfficientRMT-Net, can effectively and accurately identify various potato leaf diseases. This approach aims to overcome the limitations of traditional methods, which are often labor-intensive, time-consuming, and prone to inaccuracies due to the unpredictability of disease presentation. EfficientRMT-Net leverages the CNN model for distinct feature extraction and employs depth-wise convolution (DWC) to reduce computational demands. A stage block structure is also incorporated to improve scalability and sensitive area detection, enhancing transferability across different datasets. The classification tasks are performed using a global average pooling layer and a fully connected layer. The model was trained, validated, and tested on custom datasets specifically curated for potato leaf disease detection. EfficientRMT-Net’s performance was compared with other deep learning and transfer learning techniques to establish its efficacy. Preliminary results show that EfficientRMT-Net achieves an accuracy of 97.65% on a general image dataset and 99.12% on a specialized Potato leaf image dataset, outperforming existing methods. The model demonstrates a high level of proficiency in correctly classifying and identifying potato leaf diseases, even in cases of distorted samples. The EfficientRMT-Net model provides an efficient and accurate solution for classifying potato plant leaf diseases, potentially enabling farmers to enhance crop yield while optimizing resource utilization. This study confirms our hypothesis, showcasing the effectiveness of combining ViT and ResNet-50 architectures in addressing complex agricultural challenges.

## 1. Introduction

The Food and Agriculture Organization of the United Nations (FAO) predicts that there will be 9.1 billion people on Earth by 2050. Food consumption will rise as the population grows [1]. Meanwhile, it is difficult for nutrient levels to increase due to a lack of agriculture and the availability of clean water. At worst, agricultural crop diseases may destroy harvests and result in starvation in a nation, especially in underdeveloped countries with low wages. Plant evaluations are often carried out with the assistance of field consultants, but this job is difficult and time-consuming and requires the involvement of local professionals. It is difficult for people to analyze all crops individually, and such procedures of agronomic appraisal are seen as being unreliable [2]. Therefore, it is crucial to detect the many plant diseases precisely and quickly. In order to address issues associated with manual processes, the researchers are developing automated systems [3].

Even though many other crops exist, like tomatoes, onions, strawberries, and cherries, among others, the potato plant is among the most often eaten crops in the world. More than a billion people throughout the world consider potatoes to be a primary staple food, and they are ranked third in terms of global food production, after rice and wheat. Production exceeds 300,000 metric tons annually on a global scale, providing individuals with crucial calorie sources and minerals [4]. The potato is widely recognized as a significant non-cereal food crop and a fundamental component of the human diet worldwide. The potato is widely recognized as a fundamental component of the global diet [5]. Potatoes contribute substantially to global nutrition and are a universal source of industrial raw materials. Another work [6] focuses on examining the dietary significance of a wide range of commercial and primitive potato cultivars. The aim is to highlight that potatoes provide more than just complex carbohydrates as part of a balanced diet. Moreover, this study explores the potential of potato-based systems as viable tools and resources for promoting healthy and sustainable food production. It takes into account the challenges posed by the growing global population, climate change, and the limited availability of arable land. Typically, potatoes consist of approximately 80–70% water, 16–24% starch, and tiny amounts (4%) of protein, fat, anthocyanins, minerals, and other constituents. Despite being high in carbohydrates, potatoes also contain substantial quantities of other essential nutrients, including proteins, minerals, and vitamins [7]. The three leading exporters of potatoes are China, India, and Russia [8].

A review carried out by the UN Food and Agriculture Organization (FOA) found that the occurrence of various diseases—most of which originate from potato crop leaves and result in a 9–11% annual decrease in output—is the primary impediment to the rate of potato growth [9]. The scientific community first employed techniques from biological science and cell biology to study potato crop leaf problems [10,11]. However, these techniques have a high degree of processing complexity and need a high level of expertise [12]. Since low-income people produce most of the agriculture, farmers cannot use such expensive techniques [13]. The swift progression of object development categorization and machine vision algorithms is the foundation for the development of automated methods for agricultural disease diagnosis. More attention is being paid to image processing and machine learning (ML) investigations, and these techniques are starting to look like attractive alternatives to continuing crop infection surveillance. Current research extensively employs support vector machines (SVM) and K-Nearest Neighbors (KNN), along with other conventional machine learning predictors such as Random Forest Trees (RFT) [14], to address classification issues associated with different plant-related disorders. While the comprehension of these machine learning (ML) methodologies is very uncomplicated and necessitates only a limited quantity of data for model development, their implementation is time-consuming and heavily dependent on proficient human resources. In addition, it is common for classic machine learning information computation methods to require a compromise between the time taken for processing and the accuracy of categorization [15].

Deep learning (DL) approaches are addressing the limitations of machine learning (ML) algorithms. In the context of food security, various DL techniques, including CNN [16], RNN [17], and long short-term memory (LSTM) [18], are gaining recognition. DL algorithms can effectively calculate informative sample feature attributes without the need for subject-matter experts. These methodologies, inspired by how the human brain recognizes objects based on examples, replicate the process of object recognition and learning. DL techniques offer advantages over multispectral assessment, providing reliable results for different agricultural research tasks. Deep learning techniques [19,20,21,22] such as GoogLeNet, DenseNet, visual geometry group (VGG), and ResidualNet are extensively studied in agricultural production for tasks like grain volume measurement, plant head identification, fruit quantification, crop disorder diagnosis, and categorization. These techniques demonstrate accurate identification while minimizing computational requirements by leveraging the structural and morphological data from analyzed images [23].

**Figure 1:**
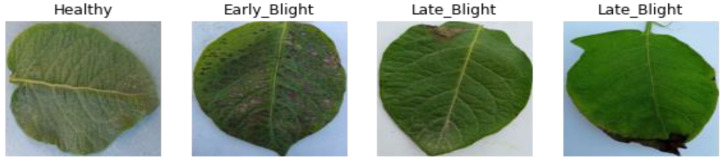
A visual example of selected image samples from each class in the dataset.

In the realm of computer vision, Vision Transformer (ViT) has just achieved a breakthrough. The utilization of global attention-based models in medical diagnosis has proven to be an effective strategy due to their capacity to comprehend the interconnectedness of global characteristics. The ViT architecture served as the fundamental framework in the study conducted by Al et al. [16]. This algorithm’s encoder comprises two separate branches, each responsible for processing a different input image: the original picture and the enhanced original image. The suggested strategy is resilient to a small quantity of training data, according to experimental results. For challenges involving multi-class classification (healthy, early, and late blight, as shown in Figure 1), Chetoui et al. [17] improved a number of ViT models. According to experimental findings, this strategy can successfully identify infected regions of potato leaves and is enhanced by employing CNN architecture to identify the diseases on potato leaf pictures. Potato-vision-transformer (PotatoViT). 

Several DL techniques [24,25,26] also showed how multi-scale data input enhanced model permanency. Studies reveal that multi-MedVit outperforms VGG16, ResNet50, and other CNN-based techniques in terms of performance. According to the literature, Trans-former is superior to CNN at classifying medical images. The parameters of the network will be significantly expanded if the Transformer structure is the sole one utilized to extract features. We suggest the ResNet Mixed with Transformer (RMT-Net) in sequence to merge the benefits of CNN with ViT.

To capture the long-distance dependency connection in the feature map and retrieve local features, RMT-Net includes Transformer, thanks to ResNet-50. To simplify processing and increase detection performance, RMT-Net introduces DWC. A novel high-precision medical image classification method for coronary pneumonia has been realized using the RMT-Net model, which has a size of just 38.5 M and detection speeds of 5.46 ms and 4.12 ms for X-ray and Potato leaf images, respectively.

### 1.1. Research Contributions

Overall, our proposed RMT-Net model addresses the challenges in image classification by leveraging the strengths of CNN, Transformer, and ResNet architectures, resulting in an effective and efficient solution for disease classification in potato leaves. In order to categorize multi-class diseases of potatoes, this research suggests a better method, EfficientRMT-Net, based on ViT. The central component of EfficientRMT-Net uses ViT as its primary framework and integrates multi-head attention modules with convolutional and residual structures. 

We can summarize our contributions in the following manner:

1. We suggest a CNN-Transformer network structure that combines the strengths of both models, allowing for the capture of both global and local features. This architecture enhances the network’s ability to understand complex patterns in the data.
2. We incorporate DWC in the final stage of the network, which helps in shrinking the number of model parameters. This parameter reduction technique improves the efficiency of the model without foregoing implementation.
3. We maintain the network architecture of ResNet while making enhancements to the feature extraction capability. By shrinking the spatial size of features and expanding the number of channels, we improve the network’s ability to extract meaningful information from the input data. Additionally, we ensure that the model size remains within an optimal range.
4. We validate the efficiency of RMT-Net as an image classification algorithm for detecting diseases in potato leaves. Through experiments and evaluations, we demonstrate that RMT-Net achieves good results in accurately identifying and classifying potato leaf diseases.
5. To control data inequity, we have employed data augmentation methods for the development of the model.

### 1.2. Paper Organization

The subsequent sections of this paper are described as follows: A literature review is described in Section 2. The circumstantials of the EfficientRMT-Net algorithm enhancement, the particular method of enhancement, and the procedure are described in Section 3; the characteristics and preprocessing of potato data are shown in Section 3.1; the performance of EfficientRMT-Net with ViT is compared and tuned in Section 3.2; and the image classification results for various categories of potato are analyzed in Section 3.3. The experimental results of EfficientRMT-Net’s successful performance were examined in Section 4. Finally, the discussions are presented in Section 5, and this paper concludes in Section 6.

## 2. Related Works

In the field of potato leaf disease detection from leaf images, various approaches have been presented. In the realm of computer vision, Convolutional Neural Networks (CNNs) have had a significant influence. LeNet, AlexNet, VGGNET, GoogLeNet, ResNet, and DenseNet are notable CNN architectures that have advanced the field. CNNs have also found application in agriculture, such as monitoring olive tree phenological responses and detecting trays with live blueberry plants [6]. Transformers, particularly in the form of self-attention-based architectures, have gained prominence in Natural Language Processing (NLP). Pretrained transformer models like BERT and GPT have achieved remarkable results in NLP tasks. Inspired by their success, the ViT model applies transformers directly to images with minimal modifications. When moderately sized datasets are used for training, like ImageNet, ViTs accuracy may be slightly lower than similarly-sized ResNet models due to the lack of certain inductive biases inherent in CNNs. However, when pretrained on larger datasets such as ImageNet, ViT approaches or surpasses the state-of-the-art performance on several picture identification benchmarks.

ViT has advantages over CNNs when trained on large datasets. It includes a more comprehensive global view and shows similarities in representations across lower and upper levels of information than ResNet in lower layers. The skip connection in ViT also plays a significant role, impacting performance and representation similarity. Overall, CNNs and transformers (such as ViT) have made significant contributions to the field of computer vision, including agriculture-related applications, and continue to advance image recognition tasks.

Several approaches have been proposed for the detection of potato leaf disease using leaf images. In the field of computer vision, the introduction of LeNet in 1989 [10] by Lecun marked the emergence of convolutional neural networks (CNNs). AlexNet [11] gained significant attention in the industry, while VGGNET [12] focused on exploring the impact of depth in CNNs. GoogLeNet [13] prioritized memory and computation usage and achieved a 6.7% error rate to win the 2014 ILSVRC Championship. ResNet [14] introduced a deep residual learning architecture to focus on the deprivation challenge, and the latest advancement, DenseNet [15], utilized dense connectivity to alleviate gradient degradation and further enhance network performance. CNNs have gradually become influential in computer vision and find application in various aspects of our lives, including agriculture. For instance, Milicevic utilized CNN algorithms such as VGG19 and ResNet50 to trace the technique of particular apparent phases of olive trees in 2020, achieving a fivefold cross-validation classification accuracy of 0.9720 ± 0.0057 [19]. Quiroz proposed an image recognition method based on CNNs in 2020 to detect trays with live blueberry plants [17].

In natural language processing (NLP), self-attention-based architectures [18], particularly transformers [19,20,21], have gained popularity. Current research suggests the use of self-attention-based architectures, particularly transformers, in natural language processing (NLP) and computer vision tasks. Several reasons are described below, which show the advantages of ViT models over other DL architectures.

1. Pretraining and fine-tuning: Transformer models and fine-tuning for specific NLP tasks.
2. Vision Transformers ViT): The success of transformers in NLP has led to their exploration in computer vision. ViT [23] is a model that utilizes a complete transformer network directly for images with minimal amendments.
3. Performance on medium-sized datasets: When trained on medium-sized datasets like ImageNet without strong regularization, ViTs accuracy is slightly lower compared to an equivalently sized ResNet (a popular convolutional neural network architecture). ViT lacks certain inductive biases inherent to CNNs, which makes it less effective when data are limited.
4. Performance on larger datasets: However, when trained on larger datasets (ranging from 14 to 300 million images) and subjected to large-scale data training, ViT outperforms or matches the latest benchmarks in image recognition. ViT achieves high accuracy on datasets like ImageNet and CIFAR-100.
5. Benefits of ViT: When applied to huge datasets, ViT outperforms CNNs. It incorporates more global data at lower levels and displays commonalities between representations acquired at lower and upper layers [24,25,26].

In [27], a thorough investigation of deep TL and convolutional neural networks (CNNs) is conducted. A pretrained model, Resnet50, is implemented for the detection and classification of plant diseases using the ImageNet dataset. The model achieves high accuracy, precision, recall, and *F*1-score on corn (maize) and potato leaf images. In [28], the authors tailored CNN to enhance accuracy while minimizing trainable parameters, computation time, and information loss. This customized model was evaluated against different standard ML and DL algorithms for potato blight classification. The results demonstrated that our model outperformed the alternatives, achieving an impressive 99% overall accuracy. It accomplished this with only 839,203 trainable parameters and a training time of just 183 s. In [29], the authors propose a multi-level deep learning model for potato leaf disease recognition. The YOLOv5 image segmentation technique is used to extract potato leaves, and a novel deep learning technique is improved for detecting blight occurring at an early stage and blight occurring at a later stage of disease. The model achieves high accuracy on a potato leaf disease dataset from the Central Punjab region of Pakistan.

The authors in [30] employed convolutional neural network (CNN) techniques to investigate the classification of five different categories of potato disease. The dataset consisted of 5000 potato images. They conducted a comparative analysis between the proposed approach and several other methods, including AlexNet, GoogLeNet, VGG, R-CNN, and TL. Whereas in [31], the authors trained distinct DL models, which include VGG16, EfficientNet B4, InceptionV3, and Inception ResNetV2, using a diverse dataset containing images of both healthy and afflicted potatoes. The performance of these models significantly exceeded the capabilities of conventional visual inspection methods. Among the evaluated models, the EfficientNet B4 model stood out with the highest level of accuracy, achieving a flawless 100% score. VGG16 followed closely with a 99% accuracy rate, Inception V3 performed at 98%, and Inception ResNet V2 achieved a commendable 94% accuracy.

A study in [32] introduces EfficientPNet, a deep learning approach using the EfficientNet-V2 network, for recognizing numerous potato leaf illnesses. The model incorporates a spatial-channel attention method and TL to address class-imbalanced samples. It achieves high accuracy in classifying potato plant leaf diseases on the challenging PlantVillage dataset. In this paper [33], the researchers selected MobileNet V2 as the base architecture due to its lightweight nature. To improve the model’s capability to capture small crop lesion features, they introduced modifications to the classical MobileNet-V2. These modifications involved containing an attention mechanism behind the pre-trained network and adding an interval convolution block for obtaining high-dimensional features. The researchers employed TL, using the PlantVillage dataset, to train the model. This dataset served as a valuable source of knowledge for the model’s learning process. As a result, the proposed procedure outperformed other methods, demonstrating superior performance. The model achieved an average identification accuracy of 97.73% across different types of potato diseases. Also in [34], the authors suggested a new approach to enhance CNN-based VGG16 for the classification of potato leaf diseases. The model leveraged the convolution layers from VGG16 [35], along with Inception and the SE block, for the classification task. To reduce model complexity, a global average pooling layer was employed, and a Squeeze and Excitation Network attention mechanism [36] was utilized to enhance feature extraction capabilities. Additionally, soft computing techniques were used for approximate calculations. Compared to traditional CNNs, our proposed model achieved an impressive classification accuracy of 99.3%.

Whereas in [37], the authors utilized ResNet-50 to extract features from leaf images and employed the modified Red Deer optimization algorithm (MRDOA) for feature selection. A deep learning convolutional neural network (DLCNN) is used for classification. The model achieves impressive results, with 99.73% accuracy, 99.78% *F*1-score for the PlantVillage dataset, and 99.68% accuracy. In contrast, the authors in [38] presented a feature selection approach (SURF) with SVM and achieved 97% accuracy. In [39], an automated crop disease detection system using a stepwise model and a CNN algorithm was developed. It accurately classifies crops and diseases (97.09% accuracy) by comparing diseased and healthy plant images. The model, adaptable for various crops, promises significant applications in smart farming, particularly for Solanaceae family crops.

In [40], the ML-based algorithms are tested, and the reported accuracy is 98% on a small dataset. The combination of CNN and KNN algorithms was utilized in this paper [41] to recognize potato plant diseases and achieved an accuracy of 90%. Whereas in [42], the authors used data augmentation techniques on healthy and diseased potato plant leaves, and they did not report the accuracy. A novel approach to identifying tomato leaf diseases using compact convolutional neural networks with transfer learning and feature selection was proposed in [43]. Their proposed pipeline outperforms previous models for tomato leaf disease classification by combining deep features from multiple CNNs and utilizing a hybrid feature selection approach. This study uses the PlantVillage dataset, which is one of the largest accessible repositories of proficiently curated plant leaf photos for the identification of diseases. The proposed framework is based on three DL models with fewer deep layers and fewer parameters, which drastically decreases the need for massive computational resources and the model construction period. This study offers a promising solution to the problem of early detection of tomato leaf diseases, which is crucial to prevent crop loss and maintain the economic significance of tomatoes. In another study [44], the authors presented an intelligent deep learning-based method to recognize nine common tomato diseases, utilizing a residual neural network algorithm for disease detection. This study investigates the impact of diverse factors, including network depth, discriminative learning rates, training and validation data split ratios, and batch sizes, on recognition performance. Experimental results reveal that the proposed method achieved the highest *F*1-score of 99.5%, outperforming most previous competing methods in tomato leaf disease recognition. A comprehensive performance comparison of five state-of-the-art CNN architectures, namely VGG16, VGG19, InceptionV3, ResNet50, and Xception, was presented in [45] for the classification of seven tomato plant diseases. The dataset used in this study consists of 18,906 images of tomato leaves, which were collected from different sources and labeled by experts. The dataset was divided into 80% for training and 20% for evaluation. The results show that all of the pre-trained models achieved similar and statistically significant performance, with accuracy ranging from 98.65% to 99.39%. The authors conclude that transfer learning is the most successful learning strategy for tomato plant disease classification. 

A framework for early detection of potato disease using deep learning strategies is implemented in [46]. This study generated a dataset of 1574 images of various diseases, which was expanded to 7870 images using data augmentation techniques. The dataset was divided into training and testing categories, and three different deep convolutional neural network architectures (AlexNet, ResNet, and GoogLeNet) were used to compare the results based on accuracy, precision, recall, and *F*1-score. This study found that ResNet gave the best performance for this particular application. In [47], the authors describe a study focused on the detection of sunn pest-damaged (SPD) wheat grains using deep learning. This study created an image acquisition mechanism to display healthy and SPD wheat grains, applied image preprocessing steps, and performed data augmentation. Two different deep learning architectures were used: transfer learning with AlexNet and a hybrid structure obtained by adding a bidirectional long short-term memory (BiLSTM) layer to the first architecture. Their proposed system found that the non-hybrid and hybrid architectures achieved 98.50% and 99.50% accuracy, respectively. The high classification success and innovative deep learning structure distinguish this study from previous ones.

The authors in [48] proposed a system aimed at classifying pepper seeds belonging to different cultivars using convolutional neural network (CNN) models. Their study used a flatbed scanner to acquire pepper seed images, followed by image preprocessing, data augmentation, and deep learning-based classification. Two approaches were proposed for classification: training CNN models (ResNet18 and ResNet50) for pepper seeds, fusing the features of pretrained CNN models, and applying feature selection to the fused features for classification using support vector machines (SVM) with different kernel functions. The proposed art found that in the first approach, the accuracies were 98.05% for ResNet50 and 97.07% for ResNet18. In the second approach, CNN-SVM-Cubic achieved up to 99.02% accuracy with the selected features.

Table 1 provides an overview of the current leading-edge information studies with limitations. Overall, these research papers highlight the success of deep learning [49] methods in identifying and classifying potato leaf diseases, providing valuable tools for early disease recognition and crop management. Overall, the passage highlights the rise of transformer models in NLP and their extension to computer vision tasks through models like ViT. While ViT initially performs slightly worse than CNNs on medium-sized datasets, it surpasses them on larger datasets when subjected to extensive training. ViT leverages the power of self-attention and transformer architectures to achieve impressive image recognition results.

## 3. Materials and Methods

**Figure 2:**
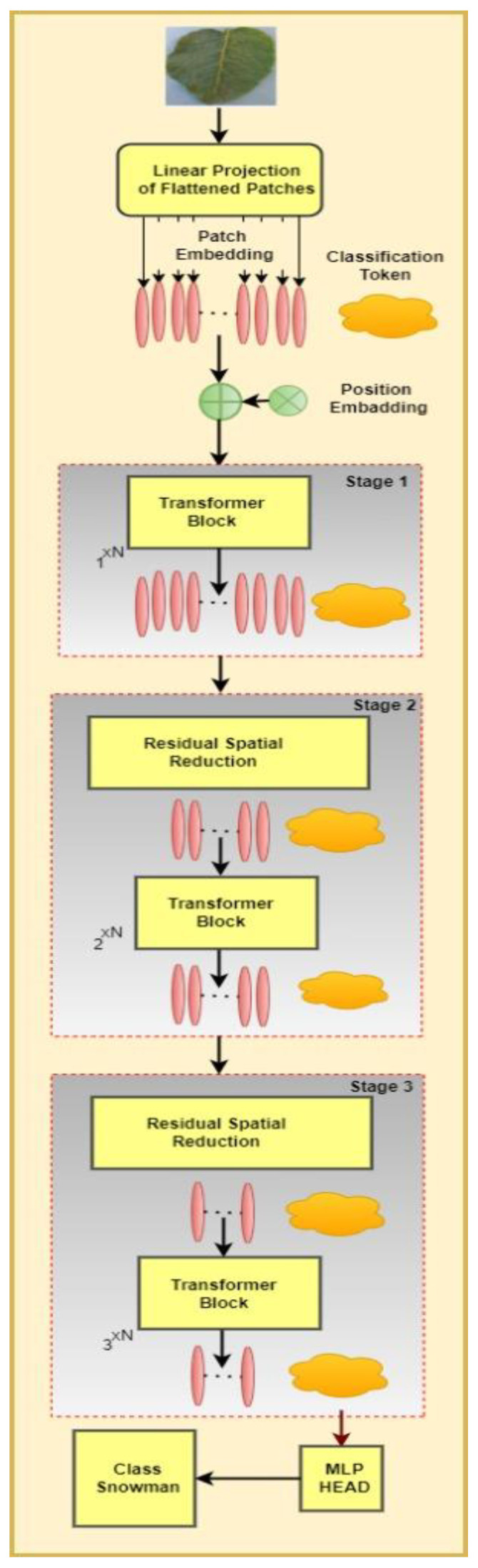
The flow of the proposed EfficientRMT-Net potato disease classification algorithm based on Resid-50 and ViT.

This paper addresses the issue of low classification accuracy in EfficientRMT-Net when applied to potato leaf images. To overcome this challenge, the authors propose a novel deep learning network called EfficientRMT-Net, which combines ResNet-50 with a Transformer architecture for improved speed and accuracy. The structure of EfficientRMT-Net is depicted in Figure 2. To improve relocation and simplification abilities, RMT-Net utilizes the four phases in the ResNet-50 backbone to allow for the extraction of features at various sizes. By stacking three-stage blocks successively with the same input resolution, the network generates hierarchical representations with varying scales.

The following paragraph describes a proposed system for potato leaf disease recognition and classification. In the first phase, the system is evaluated using the PlantVillage dataset, which contains 54,306 potato plant images. Afterwards, data augmentation techniques are used to balance the classes in the dataset, ensuring sufficient representation of healthy and diseased plants. In the second phase, enhancement of EfficientRMT-Net is performed by adding layers, which are introduced at the base of the model to portray additional complex image patterns and features, enhancing the model’s performance. Lastly, the enhanced model, called the improved EfficientRMT-Net, is trained on a large dataset of labeled potato leaf images using supervised learning. By learning from the labeled examples, the model becomes capable of classifying new images based on the identified patterns. The extra layers situated at the model’s base improve its ability to capture low-level features and patterns, leading to enhanced precision and robustness. To develop this model, techniques like TL, data augmentation, and regularization can be applied to further enhance the efficacy of the model. These techniques help improve generalization, increase the diversity of the training data, and prevent overfitting.

The passage mentions that Figure 2 illustrates the comprehensive flow of the enhanced model, while Algorithms 1 and 2 provide a detailed description of the entire procedure. These visuals likely outline the steps and processes involved in training and using the improved EfficientRMT-Net model.

Overall, the proposed system aims to enhance the recognition and classification of potato leaf diseases by improving the EfficientRMT-Net model through the addition of extra layers. The system’s potential impact lies in its ability to assist in improving crop health and productivity in potato farming.
**Algorithm 1:** Training and Inference using ResNet-50 and Vision Transformer
1Input: Training dataset (potato leaf images with corresponding labels), Test dataset (unlabeled potato leaf images)2Output: Trained hybrid model, Predicted labels for the test dataset3Initialize the ResNet-50 model and the ViT model with self-attention.4Freeze initial layers (optional) in the ResNet-50 model to preserve pre-trained weights.5Define a loss function, such as cross-entropy, in order to calculate the discrepancy between the expected and actual labels.6Decide on an optimizer, like stochastic gradient descent (SGD) or Adam, to update the model’s weights during training.7Split the training dataset into training and validation sets.8Iterate over the training set for a fixed number of epochs:9a. Initialize the cumulative loss to 0. b. Shuffle the training set. c. Divide the training set into mini-batches of a specific size.10i. Clear the gradients of the optimizer, ii. Randomly select either the ResNet-50 model or ViT model for training, iii. Forward pass the mini-batch through the selected model, iv. Calculate the loss between the predicted labels and actual labels, v. Backpropagate the loss to compute the gradients, vi. Update the model’s weights using the optimizer, and vii. Update the cumulative loss.
e. Calculate the average loss over the training set for the current epoch, f. Evaluate the model’s performance on the validation set: i. Initialize the cumulative validation loss and accuracy to 0. ii. For each mini-batch in the validation set:11- Randomly select either the ResNet-50 model or the ViT model for evaluation. - Forward-pass the mini-batch through the selected model. - Calculate the difference between the predicted labels and the actual labels. - Update the cumulative validation loss and accuracy.
iii. Calculate the average validation loss and accuracy. g. Print or record the average training and validation losses and accuracy for the current epoch.12After training is complete, select either the ResNet-50 or ViT model based on the validation performance.13Use the selected model for inference: a. Iterate over the test dataset: i. Pass each potato leaf image through the selected model. ii. Obtain the predicted probabilities for each disease category. iii. Select the disease category with the highest probability as the predicted label. b. Return the predicted labels for the test dataset.14Return the trained hybrid model and the predicted labels for the test dataset.

**Algorithm 2:** Multihead attention mechanism AlgorithmThe MultiheadAttention mechanism operates on an input tensor ‘x’ of the shape ‘(batch_size, channels, height, width)’. Here is a step-by-step breakdown of the mathematical operations involved: 1. Splitting into Heads:-The input tensor ‘x’ is split into ‘num_heads’ equal-sized chunks along the channel dimension. Each chunk represents a separate attention head.-Let us denote ‘C’ as the number of channels in ‘x’, and ‘d’; as the dimensionality of each attention head (so ‘C’ should be divisible by ‘num_heads’).-After splitting, each attention head tensor will have a shape of ‘(batch_size, C/num_heads, height, width)’.2. Attention Calculation for each Head:-Each attention head performs its own attention mechanism independently.-Let us consider a single attention head tensor ‘x_head’ of the shape ‘(batch_size, C/num_heads, height, width)’.3. Spatial Attention Mechanism:-The ‘SpatialAttention’ module calculates attention scores and attends to relevant spatial locations within each attention head tensor.-The attention score computation involves a convolutional layer and sigmoid activation:-‘att = conv(x_head)’-‘att = sigmoid(att)’-The attention scores ‘att’ are obtained and expanded to have the same shape as ‘x_head’ for element-wise multiplication.4. Element-wise Multiplication:-The attended tensor for each attention head is obtained by element-wise multiplying ‘x_head’ with the attention scores ‘att’.-‘attended_head = x_head × att’5. Concatenation of Attended Heads:-After processing each attention head, the attended head tensors are concatenated along the channel dimension.-The concatenated tensor represents the output of the MultiheadAttention mechanism.-The output tensor will have a shape of ‘(batch_size, C, height, width)’.
Exit

### 3.1. Net Framework

EfficientRMT-Net utilizes the ResNet-50 backbone with four apparent steps to obtain features on different scales, enhancing its migration and generalization capabilities. To achieve a hierarchical structure in the network and generate diverse hierarchical representations, three stage blocks are successively stacked in order to extract features using the same input resolution of varying dimensions.

To address the transformer’s inability to modify the feature map scale, patch aggregation is employed to create downsampling, enabling the hierarchical structure of the network. Before each stage, a 2 × 2 convolution with a stride of 2 is used for downsampling. The input image dimensions are 256 by 256 by 3. Following the initial downsampling in the Stem, a 128 × 128 feature map is generated, and at the end of each cycle, a dual downsampling process is executed. Following the average pooling and fully connected layers, the classification outcomes are generated. Figure 1 illustrates the EfficientRMT-Net framework, depicting the flow and components of the model.

As a fundamental developing element for processing input data, Stem can preprocess the image’s feature information, including segmentation, spatial dimension reduction, linear feature transformation, etc. Stem transforms the image $X_{\;}=R^{H\times W\times C}$ into two-dimensional image regions $X_{p}=R^{N\times (P^{2}\times C)}$, which can be viewed as $N=(HW)/P^{2}$ flattened two-dimensional sequence blocks with a dimension of P2C. Where *P* is the sequence block size and *C* is the dimension of the feature channel. Each two-dimensional sequence is subjected to a linear transformation (that is, the entirely connected layer) and compressed into a one-dimensional feature vector by Position Embedding.

The transformer architecture consists of two main sections: the encoder and the decoder. The primary components of the encoder consist of a multi-head self-attention module and a positional Feedforward Network (FFN) [19]. This addresses the issue of training deep neural networks; residual connections are used within each sub-module of the transformer. In the decoder, adjustments are made to the self-attention module to ensure that the output vectors maintain the same order. Figure 2 illustrates the structure of the transformer.

For lightweight transformer models, vision transformers (ViT) [29] and Visual Transformer (VT) [27] are commonly employed. These structures help decrease the number of model parameters while preserving performance.

In the proposed system, VIT is utilized in stage 1 to perform early global feature inference. To transform the input image into a linear input sequence, the image is divided into patches of a predetermined size. Linear embedding and position embedding are applied to each patch, and the resulting linear input sequence is fed into the standard Transformer encoder. For image classification purposes, a supplementary “classification token” is added to the start of the sequence before training. The Transformer encoder is composed of two modules: Multi-head Self-Attention (MHSA) and Multilayer Perceptron (*MLP*). Each module incorporates residual connections and applies LayerNorm (*LN*) for normalization. Equations from (1) to (4) represent the calculations performed by each component.

(1) $$Z_{0}=\left[X_{class};E;X_{p}^{2}E,\ldots ,X_{p}^{n}E\right]+E_{pos}$$

(2) $${Z_{0}}_{l}^{/}=MSA\;(Ln\left(Z_{l-1}\right)+Z_{l-1}$$

(3) $$Z_{l}=MLP\;(Ln\left({Z_{0}}_{l}^{/}\right)+{Z_{0}}_{l}^{/}$$

(4) $$y=Ln\left({Z_{\;}}_{l}^{0}\right)$$

where $E\;\epsilon R^{D\times (P^{2}\times C)}$, $E_{pos}\;\epsilon R^{D\times (N+1)}$. In the context of the given information: In CNN, each layer’s features exhibit locality, a two-dimensional neighborhood structure, and shift-invariance. It operates on a set of real numbers, unless otherwise specified. In VIT, the self-attention layer captures global features, while only the MLP layer exhibits locality and shift-invariance. Hence, VIT is utilized in Stage 1 for global feature inference. Unlike CNN, VIT emphasizes global features and rapidly extracts network-beneficial features in the early stages. As the network deepens, the number of features gradually increases. To achieve global feature modeling and reduce network parameters, the VT module is employed in Stage 2. These operations can be represented using Equation (5).

(5) $$T={HW}_{SoftMax}{\left({XW}_{A}\right)}^{T}X$$

where, $W_{A}\epsilon R^{C\times L}$ forms semantic groups from *X*, *SoftMax*(⋅) is the softmax activation function, and *X* represents the feature map. For the input feature map *X*, VT uses point convolution to map each pixel $X_{p}\epsilon R_{c}$ in the feature map into L groups, and then uses spatial pooling to obtain tokens. To address the sparsity of high-level semantic information and avoid computational waste, VT adopts a concatenation approach. All layers of the VT module utilize the output of the EfficientRMT-Net layer as input, gradually refining the visual tokens. This allows for the modeling of the relationship between semantics and the transformer. The transformation is achieved by converting all tokens into weights through Softmax and multiplying them with the original feature map X, resulting in the reassigned attention map. Equation (6) is employed to incorporate the relationship between semantics and the transformer.

(6) $${T^{\;}}_{out}^{’}=T_{in}+{HW}_{SoftMax}(T_{in}K){\left(T_{in}Q\right)}^{T}T_{in}$$

(7) $$T_{out}^{\;}={T^{\;}}_{out}^{’}+{\sigma \;({T^{\;}}_{out}^{’}F_{1})}_{\;}F_{2}$$

**Figure 3:**
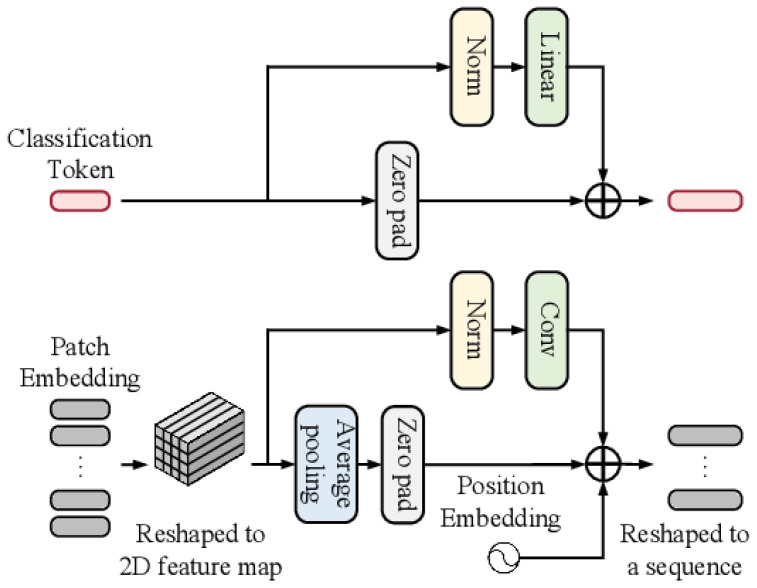
Architecture of the residual connection with ViT.

In Stage 3 of the proposed system, both MHSA (Multi-head Self-Attention) and LMHSA (Lightweight Multi-head Self-Attention) modules are represented by Equation (8).

(8) $${Attentation}_{light-weight}\left(Q,K,V\right)=Softmax\left(\frac{QK^{T}}{\sqrt{d_{k}}}+B\right)V$$

where bias B ∈ R is a learnable parameter. The understood comparative positional bias can also be transferred to different sizes by bicubic interpolation (Figure 3).

### 3.2. Loss Function (LF)

The loss function (LF) plays a crucial role in evaluating the performance of models. It is utilized by networks during automated learning to assess their predictions against the actual values in large datasets. The important goal of the LF is to assess the discrepancy between the calculated and real quantities. During the model training process, the LF is iteratively adapted to minimize the error and achieve a robust fit to the data. The ultimate aim is to obtain a well-fitting LF that accurately captures the connection or correlation between the forecasted and observed values.

### 3.3. Fine-Tune Network

Fine-tuning hyperparameters is an essential step in optimizing the performance of a model. Here are some commonly tuned hyperparameters as defined in Table 2 for the combination of ResNet-50 and ViT architectures with self-attention for the classification of potato leaf diseases:

1. Learning Rate: Fine-tuning the learning rate can significantly impact the training process. A higher learning rate may lead to faster convergence, but it could also cause instability or overshooting. Conversely, a lower learning rate might ensure more stable training but could require a longer time to converge. It is advisable to experiment with different learning rates, such as 0.1, 0.01, and 0.001, and observe the impact on the model’s performance.
2. Number of Epochs: The total number of training dataset iterations that the model goes through during training is determined by the number of epochs. Increasing the number of epochs may allow the model to learn more complex patterns, but it could also lead to overfitting. On the other hand, using too few epochs may result in an undertrained model. It is important to strike a balance by monitoring the validation performance and adjusting the number of epochs accordingly.
3. Batch Size: The quantity of training samples handled in a single forward and backward pass is referred to as the batch size. More memory may be needed; however, quicker training may result from a larger batch size. A smaller batch size may allow better convergence and generalization, but it could also slow down the training process. Experimenting with different batch sizes, such as 16, 32, and 64, can help identify an optimal value.
4. Regularization Techniques: Overfitting may be avoided, and generalization can be strengthened with the use of regularization methods like dropout, weight decay, or batch normalization. Fine-tuning the regularization strength or adjusting the dropout rate can be beneficial. For instance, try dropout rates of 0.3, 0.5, or experiment with different weight decay values to strike a balance between regularization and model performance.
5. Data Augmentation: Data augmentation techniques, such as random crops, flips, rotations, and brightness adjustments, can improve the model’s capability to generalize. Fine-tuning the augmentation parameters, such as the degree of rotation or the range of brightness adjustments, can impact the model’s performance. Experiment with different augmentation configurations to find the optimal settings for the potato leaf disease classification task.
6. Transformer Layers: In the ViT architecture, the number of transformer layers can affect the model’s representation capacity and computational efficiency. Adjusting the number of layers, such as 6, 12, or 24, can influence the trade-off between model complexity and performance. Experiment with different layer configurations to find the optimal balance.
7. Attention Heads: The number of attention heads in the ViT model affects the attention mechanism’s granularity and capacity for capturing dependencies. Fine-tuning the number of attention heads, such as 4, 8, or 16, can impact the model’s performance. Experiment with different head configurations to find the optimal setting for the potato leaf disease classification task.

## 4. Experimental Results

The experimental implementation of the proposed RMTNet model utilized the TensorFlow framework [29], together with the Keras open-source libraries and the Python programming language. The Adam optimizer was employed with a default learning rate, and the categorical cross-entropy loss function was used for training. The experiments for the proposed art were conducted using a Lenovo laptop equipped with an 11th generation Intel Core i7 processor running at 2.40 GHz. The laptop had 16.0 GB of memory and was working on the Windows 10 operating system, specifically the 64-bit version.

The dataset that was utilized to train and assess the classification outcomes of our suggested method for categorizing various potato plant leaf diseases is briefly described in this section. We also outline the performance measures utilized to evaluate the efficiency of our approach. Furthermore, we conducted a comprehensive evaluation with numerous other models to demonstrate the superior performance of our methodology. 

### 4.1. Dataset Acquisition

In our study, we evaluated the capacity of our framework to be recognized using a standard dataset known as the PlantVillage repository [38]. This dataset is freely available online and is commonly used for simulating and evaluating models. The 54,306 photographs in the PlantVillage collection are part of a large library of plant leaf photos. For the purpose of performance assessment, we have selected a subset of this dataset that is consistent with our focus on the exclusive classification of leaf diseases in potato crops. The disease types included in the PlantVillage dataset are listed in Table 3.

**Figure 4:**
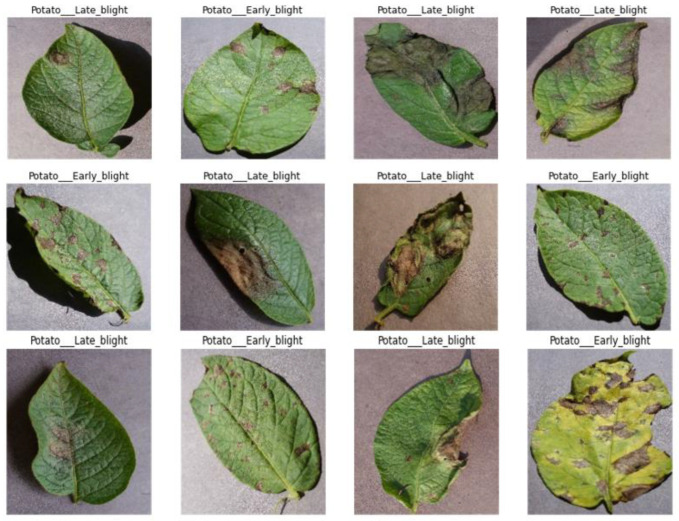
An illustration of a sample dataset.

We chose this dataset for performance testing because it includes diverse samples with variations in leaf mass, structure, size, orientation, and infected regions. Additionally, the dataset exhibits various distortions, such as clutter, blur, intensity variations, and color variations. Figure 4 provides a visual representation of a few samples from the PlantVillage dataset. Please note that for a more detailed analysis of the performance evaluation and results, additional information or references would be required.

### 4.2. Data Augmentation

The PlantVillage dataset, which contains photos of potato leaf diseases, was used to train, verify, and evaluate the effectiveness of our suggested deep learning model. Images of early and late blight as well as healthful potato leaf conditions were included in this dataset. The resolution of every picture in the collection was (256 × 256) pixels. While the pictures of early and late blight showed the two different phases of potato leaf disease development, the photographs of healthy potato leaves showed leaves in a normal and disease-free condition. To represent the three classes in the dataset, we gave the indices 0, 1, and 2. Table 4 displays the division of the total number of photos in the dataset across the different categories.

It is noteworthy to emphasize that the number of photos depicting healthy potato leaves was lower than that of the other two groups that represented potato blight. We used data augmentation to correct this imbalance in the dataset. To achieve this, 10 images of robust potato leaves were chosen at random, and ten copies of each image were made. This procedure was carried out five times. After we balanced the dataset, the final table, Table 3, displays the total number of photos in each class. At first, there were 600 photos of healthy potato leaves and 1200 photos of early and late blight in each group. After data augmentation, 3600 photos of potato leaves in good health as well as 3600 photographs of early and late blight were included in each category.

To prevent overfitting and train the model effectively, we regularized the data and expanded the size of the training set. Data augmentation techniques were applied, including rotating the photos within a range of −20 to +20 degrees, shearing between −40 and +40 degrees, and shifting the width and height within a range of 0.2. Figure 4 illustrates the image creation of the augmentation procedure.

### 4.3. Performance Metrics

A confusion matrix is a table of size n × n that is used to record the predictions made by a classifier with n classes. It provides information about the performance of the classifier by comparing the predicted classes with the actual classes. The confusion matrix includes elements such as True Positive (*TP*), True Negative (*TN*), False Positive (*FP*), and False Negative (*FN*).

We calculate the accuracy (*Acc*) in order to assess the overall performance of the classifier. The proportion of properly identified samples relative to the total number of samples is known as accuracy. It can be computed using the following formula, as described in Equation (9).

(9) $$Accuracy\;\left(ACC\right)=TP+TN/TP+TN+FP+FN\;\times \;100\%$$

Precision (*P*) is a metric that measures the proportion of samples predicted to be in the positive category that are actually in the positive category. It provides an indication of the classifier’s ability to correctly identify positive samples. Precision is calculated using the formula in Equation (10). Precision is expressed as a percentage and represents the accuracy of positive predictions out of all positive predictions made by the classifier. It is an important metric for tasks where the focus is on minimizing false positives and ensuring the correctness of positive predictions, as calculated by Equation (10).

(10) $$Precision\;\left(P\right)=\frac{TP}{TP+FP}\times 100\%$$

The fraction of samples that really belong to the positive class and are correctly recognized as positive by the classifier is measured by recall (*R*), also known as sensitivity or the true positive rate. It evaluates the classifier’s ability to identify all positive samples. Recall is calculated using the formula in Equation (11). Recall is expressed as a percentage and represents the proportion of true positive predictions out of all actual positive samples. It is an important metric when the focus is on minimizing false negatives and ensuring the completeness of positive predictions. A high recall indicates that the classifier is effectively capturing most of the positive samples in the dataset.

(11) $$Recall\;\left(R\right)=\frac{TP}{TP+FN}\times 100\%$$

A statistic called the *F*1-score (*F*1) combines recall and accuracy to provide a single evaluation of the classifier’s effectiveness. It provides a comprehensive assessment by taking into account both accuracy (ability to precisely identify positive samples) and recall (capacity to include all positive samples). The *F*1-score is calculated using the formula in Equation (12). The *F*1-score is expressed as a percentage and ranges from 0 to 100%. A high *F*1-score indicates that the classification method is effective in achieving a balance between precision and recall. It is especially useful when precision and recall may contradict each other, such as in imbalanced datasets or when there is a trade-off between minimizing false positives and false negatives. By considering both precision and recall, the *F*1-score provides a comprehensive evaluation of the classifier’s overall performance.

(12) $$F1=2\times \frac{P\times R}{P+R}\times 100\%$$

Specificity, also known as the false positive rate (*FPR*), measures the proportion of negative samples that are misclassified as positive out of all actual negative samples. It is the complement of the true negative rate. Specificity is calculated using the formula in Equation (13).

(13) $$FPR=\frac{FP}{FP+TN}\times 100\%$$

The ROC curve (Receiver Operating Characteristic) and the PR curve (Precision-Recall curve) are evaluation metrics commonly used for assessing the performance of classifiers. Both curves provide insights into the trade-off between true positive rate (TPR) and false positive rate (*FPR*), or precision and recall, respectively. The area under the ROC curve (AUC) is a popular metric derived from the ROC curve. It represents the overall performance of the classifier, with values closer to 1 indicating better classification performance. AUC measures the ability of the classifier to distinguish between positive and negative samples across different thresholds.

### 4.4. Experimental Results

During the first model assessment phase, we examined the performance of our suggested method by analyzing the data per class in order to gauge the model’s ability to classify different types of potato plant leaf abnormalities. We used a variety of performance measures to assess the performance, and the results are shown below. In order to confirm the experimental results, we also sought the assistance of a plant pathologist.

**Figure 5:**
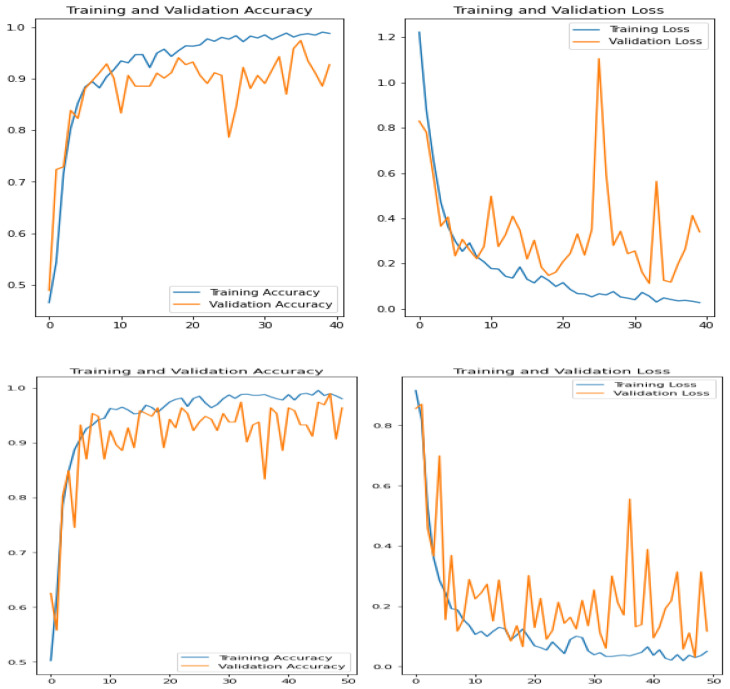
Loss/Validation accuracy curves for the proposed model.

The accuracy and loss during training and validation of our suggested model are shown in Figure 5. The Train_acc and Train_loss curves clearly show a fast fall with training, suggesting that the suggested model achieves good training results quickly and stays stable. The suggested model achieves 99.64% Train_acc and 0.0132 Train_loss on the image dataset by the 50th epoch and 99.87% and 0.0102 on the Potato leaf image dataset. These results demonstrate that, when it comes to training performance on both datasets, our suggested model performs better than the alternative models mentioned in Table 2. The RMT-Net model’s stability and exceptional performance are confirmed by the curve patterns and magnitudes. Crucially, it should be mentioned that the experimental findings were confirmed by a qualified plant pathologist, which increased the assessment process’s legitimacy.

### 4.5. Training Process

In order to visually aid in understanding the RMT-Net model’s training process, we chose to show the loss values from the first 100 training epochs on photos of potatoes. Table 5 displays the changes to the Train_acc and Train_loss variables.

**Figure 6:**
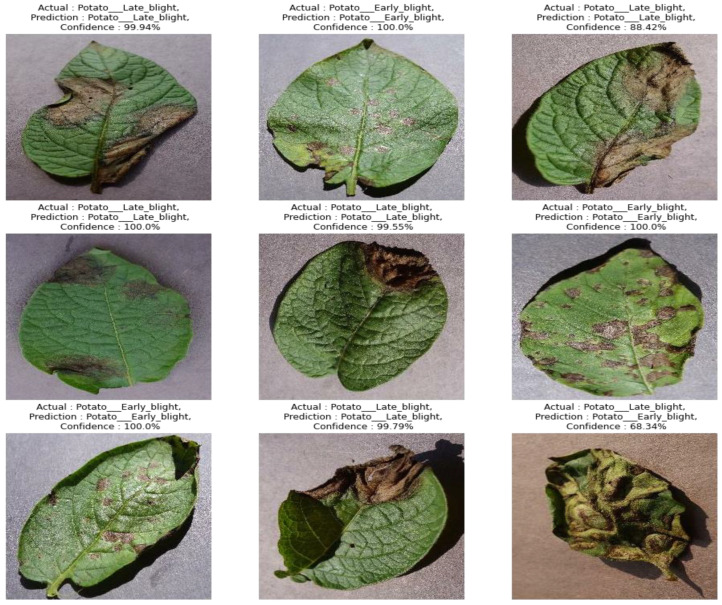
Sample results were recognized by the proposed system.

As the training progresses, it is evident that the Train_acc and Train_loss curves of the proposed model decrease rapidly, indicating effective learning within a short training period while maintaining stability. After 50 epochs, the proposed model achieves a Train_acc value of 99.64% with a Train_loss value of 0.0132 on the dataset and 99.87% with a Train_loss value of 0.0102 on the Potato leaf image dataset. These results demonstrate that the proposed model attains the best training performance on the Potato leaf image dataset. When compared to the other models listed in Table 4, the proposed model exhibits superior training results on Potato leaf image datasets. The curve’s trend and amplitude are exceptional, further validating the stability of the proposed model (Figure 6).

### 4.6. Ablation Study

An ablation study is a key part of the experiment process for models like the EfficientRMT-Net to show that the proposed Conv-ViT framework works. This framework combines advanced architectures such as Vision Transformers (ViT) and convolutional neural networks (CNNs). In such a study, key components of the model are systematically removed or altered to understand their individual contributions to the model’s overall performance. In the case of EfficientRMT-Net, this would mean trying out different setups by turning off or changing parts like the ViT layers, depth-wise convolution (DWC), or the enhanced ResNet features. Each variant of the model is then evaluated against standard metrics to ascertain the impact of these components on the model’s ability to accurately classify and detect features in the input data. Table 6 represents the impact of different architectures on the model’s performance for recognizing potato leaf diseases.

The ablation study for the EfficientRMT-Net model demonstrates the significance of integrating various architectures and techniques to enhance model performance for classification tasks. Table 6 compares different model configurations over 50 epochs on several metrics: Sensitivity (SE), Specificity (SP), Accuracy (ACC), Precision (PR), and *F*1-score. The standalone models, including EfficientNet, Vision Transformers (ViT), depth-wise convolution (DWC), and ResNet, show varying degrees of effectiveness, with their accuracy ranging from 76% to 84.6%. The combination models, such as ViT with ResNet and EfficientNet with ResNet, also display varied results, with accuracy peaking at 84.6% for the ViT and ResNet combination. However, the most notable finding is the performance of the EfficientRMT-Net, which combines EfficientNet, ViT, DWC, and ResNet. This configuration shows a substantial improvement in all metrics, achieving an impressive accuracy of 97.6%, along with high scores in sensitivity (94%), specificity (96%), precision (94.12%), and an *F*1-score of 96.2%. These results underscore the synergistic effect of integrating these different architectures and techniques, highlighting that the combined approach in EfficientRMT-Net significantly outperforms the individual models and their simpler combinations. This comprehensive integration effectively captures both global and local features, enhances feature extraction, and maintains efficiency, leading to superior performance in classification tasks.

### 4.7. Computational Efficiency

To verify whether the proposed EfficientRMT-Net meets the required reasoning speed, a comparison experiment was conducted against four other models, and the results are presented in Table 4. ResNet-50: Training ResNet-50 on a large dataset can take several hours to days, depending on the hardware and optimization techniques used. Vision Transformer: Training ViT models can also take a significant amount of time, especially with large-scale datasets. The training time can range from several hours to days or even weeks.

The table displays the detection speeds for each image on potato image data for ResNet-50, VGGNet-16, i-CapsNet, and Inceptionv-3 models, as well as the EfficientRMT-Net model. The detection speeds for each model are reported as 12.24 ms, 10.09 ms, 8.58 ms, 6.06 ms, and 5.46 ms, respectively. It is evident that the detection speed of the EfficientRMT-Net model is significantly faster than that of the other networks. For instance, EfficientRMT-Net is 55.4% faster than ResNet-50, 45.9% faster than VGGNet-16, 36.4% faster than i-CapsNet, and 9.9% faster than Inceptionv-3.

Similarly, on potato leaf image data, the detection speeds are reported as 10.37 ms, 7.83 ms, 5.79 ms, 4.23 ms, and 4.12 ms for the ResNet-50, VGGNet-16, swin, GoogleNet, and EfficientRMT-Net models, respectively. Here, RMT-Net demonstrates a 60.3% improvement in detection speed compared to ResNet, a 47.4% improvement compared to VGGNet-16, a 28.8% improvement compared to swin, and a slight 2.6% improvement compared to GoogleNet.

In addition to the reduction in model size, there are two other factors contributing to the improved speed of EfficientRMT-Net. Firstly, the overall structure of EfficientRMT-Net differs from the classic transformer as it adopts a pyramid structure, which gradually decreases the spatial dimension, resulting in enhanced computational efficiency. Secondly, in terms of micro-design, EfficientRMT-Net employs a lightweight self-attention structure and incorporates DWC in the last stage of the network to further reduce the model’s complexity. These design choices contribute to the high computational efficiency of the EfficientRMT-Net algorithm.

It is important to note that these time estimates are approximate and can vary based on various factors. The actual computational time can be influenced by the complexity of the model architecture, the size of the input images, the available computational resources (CPU or GPU), and the optimization techniques implemented. Therefore, it is recommended to benchmark the models on your specific hardware and dataset to obtain more accurate time estimates.

### 4.8. Comparison to the Related Literature

This paper compares the performance of Efficient-RMT-Net with other classification models, as presented in Table 5. The numbers in brackets in the third column represent the number of categories (2, 3, or 4). The results demonstrate that RMT-Net achieves superior classification performance compared to other models in both the four-classification of potato leaf images and the two-classification of Potato leaf images. Specifically, RMT-Net achieves an accuracy rate of 97.65% in potato plant leaf classification.

**Figure 7:**
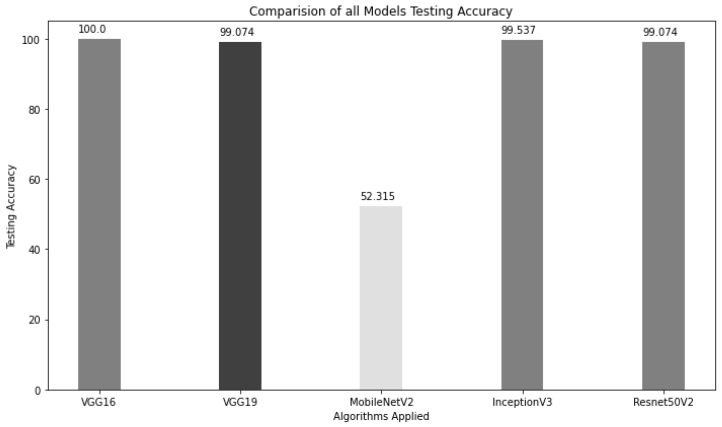
Standard DL-based models are used to recognize potato leaf diseases.

RMT-Net, proposed in this paper, combines ResNet-50 and Transformer to leverage the strengths of both architectures. By using Transformers, EfficientRMT-Net captures long-distance dependencies, while CNN is employed to extract local features. The model also incorporates DWC to reduce computation and a stage block structure to enhance scalability, receptive field, and transferability. In comparison to other classification models, EfficientRMT-Net demonstrates excellent performance in terms of classification accuracy, model size, and detection speed (Figure 7).

Potato leaf images, as well as leaf images, can provide more detailed information about diseases, thereby enriching the dataset. Consequently, further research is warranted to develop adaptive networks with higher accuracy and faster detection capabilities. The accuracy of convolution-based object detection methods heavily relies on the choice of feature extraction backbone network. A well-designed backbone network is crucial for achieving excellent object detection performance. This article provides a detailed introduction to classic backbone networks [1]. Moreover, researchers are continuously exploring different techniques, methods, and combinations to improve CNN-based backbone networks and detectors. While the application of pure Transformers in computer vision has garnered less attention, recent research on target detection has predominantly focused on Transformers.

**Figure 8:**
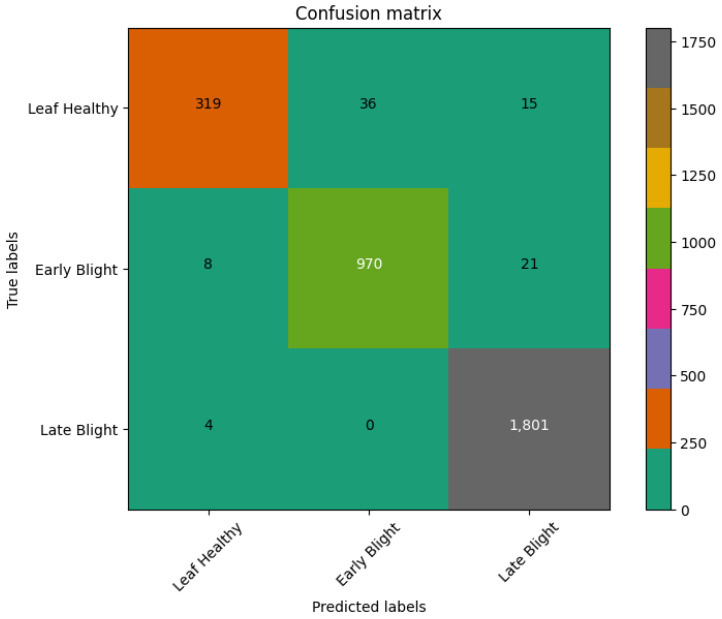
Proposed confusion matrix for three classes (leaf healthy, early blight, and late blight) using the PlanVillage dataset.

**Figure 9:**
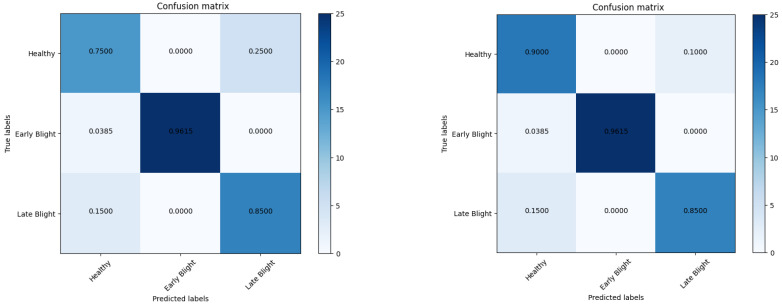
Confusion matrix for three classes (leaf healthy, early blight, and late blight) by utilizing the methods of Paul [23] and Olawuyi [27] on the PlanVillage dataset.

Table 7 represents comparisons with recent state-of-the-art studies such as Paul [23]-EfficientNet, Olawuyi [27]-Transfer, Nazir [28]-EfficientPNet, Chen [29]-Cnn, Barman [30]-DL, and Mahum [31]-Efficient for potato disease classification. Based on this table, the proposed system achieved better performance. Moreover, the confusion matrix obtained in Figure 8 and Figure 9 by the proposed art further endorsed its significant performance in detecting three classes.

To facilitate the model’s generalizability, it was trained on datasets apart from the potato leaf dataset. In order to accomplish this, we evaluated the efficacy of the proposed method using Tomato and Grape leaves from the publicly accessible dataset PlantVillage (PV) and noticed promising results, as stated in Table 8. The Tomato dataset was derived from the PV dataset. It consists of 18,160 photos obtained from the PV dataset of 9 damaged and 1 healthy category of tomato leaves. Each image has a resolution of 256 × 256. While the Grape dataset has 4062 instances of 256 × 256 photos depicting four distinct categories of grape leaves, including three diseased categories and one healthy category. 

## 5. Discussions

In presenting the EfficientRMT-Net model for potato plant leaf disease classification, it is crucial to acknowledge not only its strengths but also its limitations, particularly concerning different cultivation conditions and environmental factors. While the model demonstrates exceptional accuracy and efficiency, its performance across varied potato cultivation conditions remains an area needing further exploration. One significant limitation is the model’s applicability in diverse agricultural settings. Potato crops are cultivated worldwide, subject to a myriad of climatic conditions and agricultural practices. These variables can introduce significant differences in leaf appearance, disease manifestation, and overall plant health. The current dataset, predominantly focused on specific types of potato species and diseases, might not fully encapsulate this diversity. Consequently, the model’s ability to generalize across different potato cultivation conditions is yet to be thoroughly tested.

Another aspect to consider is the model’s classification performance under various climatic and lighting conditions. Environmental factors such as humidity, temperature, and lighting can significantly impact the visual appearance of leaf diseases. For instance, the same disease may present differently under bright sunlight compared to overcast conditions, potentially leading to classification challenges. Moreover, the quality of images captured under different lighting conditions could affect the model’s accuracy. Images with poor lighting or high variability in exposure might not be classified as effective, raising concerns about the model’s robustness in real-world scenarios. Future research should aim to expand the dataset to include a broader range of potato species cultivated under diverse environmental conditions. This would not only enhance the model’s generalizability but also its practical utility for farmers worldwide. Additionally, experimenting with images taken under various climatic and lighting conditions could help refine the model, ensuring consistent performance regardless of external environmental factors.

In this research, we presented EfficientRMT-Net, a deep learning solution for the classification of potato plant leaf diseases. Our results demonstrate that EfficientRMT-Net outperforms other classification models in terms of accuracy, model size, and detection speed. This highlights the effectiveness and efficiency of our proposed approach in addressing the challenges associated with potato plant diseases. One of the major benefits of EfficientRMT-Net is its facility to extract high-level indicators of infected regions and establish important relationships between tokens. This is achieved through the integration of global self-attention mechanisms and residual blocks, which enable the model to capture both global and detailed features of potato leaf images. By leveraging an end-to-end learning mechanism, EfficientRMT-Net robustly recognizes and classifies different types of potato maladies.

Our experiments on the PlantVillage dataset demonstrate the efficacy of EfficientRMT-Net in distinguishing potato illnesses, even from warped images. The model exhibits high classification accuracy, indicating its ability to effectively distinguish between healthy leaves, leaves with early blight, and leaves with late blight. This is particularly important in mitigating the detrimental impact of these diseases on potato crop yield and farmers’ profits. While EfficientRMT-Net shows promising results, there are some limitations to consider. Firstly, the dataset used in this study is limited to potato leaf images, which may affect the generalizability of the model to other plant species. Additionally, the availability of distorted images in the dataset could have introduced some challenges in classification. Table 6 represents differences with recent state-of-the-art studies such as Paul [23]-EfficientNet, Olawuyi [27]-Transfer, Nazir [28]-EfficientPNet, Chen [29]-Cnn, Barman [30]-DL, and Mahum [31]-Efficient for potato disease classification. Based on this table, the proposed system achieved better performance.

Future research should focus on developing the dataset to include a broader range of plant species and investigating methods to improve the model’s robustness to image distortions. As a future objective, we plan to develop a second ensemble model by integrating explainable AI techniques and the EfficientRMT-Net architecture. This will allow for a deeper interpretation of the management process of the model and provide interpretable results, which can be valuable for plant pathologists and researchers. Furthermore, we aim to test our approach on more challenging datasets to further evaluate its performance and investigate its applicability in real-world scenarios. In conclusion, our study demonstrates the effectiveness of EfficientRMT-Net in the classification of potato plant leaf diseases. The model exhibits superior performance compared to other classification models, offering accurate and efficient detection capabilities. We believe that the proposed approach can contribute to more efficient and accurate potato classification by aiding in the conservation of potato germplasm diversity and supporting farmers in minimizing crop losses.

### 5.1. Advantages of the Current Approach

The acquisition of potato species images can be challenging, emphasizing the importance of a deep learning algorithm that can generalize well and identify different potato species effectively. The EfficientRMT-Net algorithm enhances the ViT algorithm by combining the convolutional residual mechanism with the multi-head attention mechanism on a dataset of potato species images. Through a comparative analysis of EfficientRMT-Net, ViT, ResNet, and VGG16 on a potato dataset, it was concluded that EfficientRMT-Net outperforms both ViT and CNNs.

ViT has been found to lack certain inductive biases, such as locality, two-dimensional neighborhood structure, and translation equivariance, especially on small-scale datasets, which can be effectively captured by CNNs. However, pretraining ViT on large amounts of data allows it to overcome the limitations of inductive bias present in CNNs and achieve superior performance. The residual mechanism, proposed by He et al. in 2016, addresses the degradation problem in deep CNNs and enhances the convolutional structure.

In this paper, the ViT algorithm is improved with the proposed EfficientRMT-Net algorithm, which demonstrates superior performance compared to both ViT and CNNs on the potato dataset. Even with a reduced number of training samples, EfficientRMT-Net still outperforms ViT. This suggests that the convolutional and residual mechanisms compensate for the inductive bias that ViT struggles to learn on small-scale datasets, making ViT applicable to classification tasks even with limited training samples.

Potato species exhibit significant variations and are distributed worldwide, making their classification and identification a challenging task that requires expertise and substantial resources. The average classification accuracy of EfficientRMT-Net reaches 90.21%, surpassing CNNs such as ResNet18, VGG16, Xception, and DenseNet121. The proposed EfficientRMT-Net algorithm can aid potato experts in conducting more efficient and accurate potato classification and identification, which is crucial for the preservation of potato germplasm diversity. Although the team faced limitations in collecting a limited number of images due to resource constraints, future research aims to gather more diverse data types, further improve the algorithms, and deploy models and data on servers to support potato image classification and other image classification applications. A list of advantages of the proposed EfficientRMT-Net compared to other machine learning, deep learning, and ViT techniques:

(1) EfficientRMT-Net achieves an accuracy of 97.65% on the image dataset and 99.12% on the Potato leaf image dataset, demonstrating its superior classification performance.
(2) The model efficiently detects and classifies potato leaf diseases, making it a time-saving and cost-effective solution for farmers.
(3) EfficientRMT-Net combines the strengths of ViTs and ResNet-50 for feature extraction, improving its ability to capture meaningful information from images.
(4) The use of DWC helps reduce computational requirements, making it more efficient for real-time applications.
(5) The stage block structure enhances scalability, enabling the model to handle various datasets and potentially adapt to different plant diseases.
(6) EfficientRMT-Net’s design allows for better TL, which can be beneficial when applying the model to different tasks or domains.
(7) The model is trained and evaluated on custom datasets, which means it may be adjusted for certain uses or regions, improving its adaptability.
(8) EfficientRMT-Net demonstrates robustness in handling distorted samples, which is crucial for real-world scenarios where image quality may vary.
(9) By efficiently identifying and managing potato leaf diseases, farmers can potentially increase crop yield and save money on resources and treatments.
(10) The model can be integrated into existing agricultural systems and tools, making it accessible and practical for farmers.

### 5.2. Future Works

In our current research, we have utilized publicly available datasets to develop and test EfficientRMT-Net. While these datasets provided a controlled environment for initial validation, we acknowledge that they may not fully represent the complexity and variability encountered in real-world settings. Specifically, outdoor images of potato leaves can indeed present additional challenges such as varying lighting conditions, background noise, and distortions caused by natural elements like wind or rain. Your observation highlights a crucial aspect of our model’s practical application: its ability to maintain accuracy and reliability in less-than-ideal conditions, which are commonplace in outdoor agricultural settings. To address this, we propose the following steps in our future research:

(1) We plan to augment our dataset with images captured directly from potato fields. This will include photographs taken under various lighting conditions, at different times of the day, and in diverse weather scenarios.
(2) Recognizing that outdoor images may contain more noise and distortion, we will focus on enhancing the model’s preprocessing and feature extraction capabilities to better handle these irregularities.
(3) We will conduct extensive testing with this expanded dataset to assess and improve the model’s robustness against real-world image variations.
(4) To ensure the practical applicability of our model, we intend to collaborate with agricultural experts and farmers to gather and validate real-world images.

We believe these steps will significantly contribute to validating the model’s performance in real-world conditions, thereby enhancing its usefulness for practitioners in the field of agriculture.

## 6. Conclusions

The EfficientRMT-Net framework proposed in the passage offers a promising solution for detecting and classifying foliar diseases in potato plants, particularly early and late blight. The model combines the ResNet-50 and Transformer architectures, utilizing Transformers for capturing long-range dependencies and CNN for extracting local features. With the inclusion of DWC, a stage block structure, and residual blocks, the model demonstrates outstanding performance in terms of classification accuracy, model size, and detection speed. The experimental results show that EfficientRMT-Net achieves high accuracy rates of 97.65% and 99.12% on different datasets, outperforming other models. Additionally, the model has a relatively compact size of 38.5 M, and the detection speeds for potato disease and Potato leaf image are 5.46 ms and 4.12 ms per image, respectively.

Overall, the EfficientRMT-Net framework proves to be efficient in accurately classifying potato plant leaf diseases and dealing with distorted samples. By implementing this technique, farmers have the potential to increase their yield while saving money by effectively detecting and addressing foliar diseases in a timely manner.

## Figures and Tables

**Table 1:** A table summarizing the comparisons of state-of-the-art methods for identifying multi-class diseases of potato plants.

Cited	Purpose	Technique	Results	Limitations
[27]	Disease classification using Resnet50	Deep transfer learning with CNNs	High accuracy, precision, recall, and *F*1-score	Not explicitly mentioned
[28]	Recognizing potato leaf disorders	EfficientNet-V2 with the spatial-channel attention method	High accuracy, class-imbalanced handling	Not explicitly mentioned
[29]	Potato disease identification using MobileNet V2	Modified MobileNet-V2 with attention and octave blocks	Average accuracy of 97.73%	Not explicitly mentioned
[30]	Potato disease detection using a self-built CNN (SBCNN)	Custom CNN on augmented and non-augmented datasets	High validation and training accuracy	No comparative analysis with other models
[31]	Potato leaf disease recognition using YOLOv5	YOLOv5 image segmentation and novel deep learning	High accuracy on the Central Punjab dataset	Limited focus on a specific region’s dataset
[32]	Classification of five potato disease categories	CNN-based approach, comparative analysis with others	Not specified	Not explicitly mentioned
[33]	Multi-model training for healthy and afflicted potatoes	VGG16, EfficientNet B4, InceptionV3, and Inception ResNetV2	High accuracy with EfficientNet B4	No comparative analysis with other datasets or models
[34]	Enhancing CNN-based VGG16 for potato blight classification	Customized CNN, compared with other algorithms	99% overall accuracy, reduced parameters	No comparative analysis with other datasets or models
[36]	Improved CNN-based VGG16 for potato leaf disease classification	CNN with VGG16, SE block, and soft computing	Impressive 99.3% classification accuracy	Not explicitly mentioned
[37]	Feature selection and classification using ResNet-50	MRDOA and DLCNN	High accuracy, *F*1-score	Not explicitly mentioned
[38]	Feature selection with SVM for disease classification	SURF feature selection with SVM	Achieved 97% accuracy	No comparative analysis with other models
[39]	CNN-based classification with pre-trained models	Five pre-trained models, achieved 97.07% accuracy	97.07% accuracy	No comparative analysis with other models
[40]	ML-based algorithms for disease classification	Machine learning algorithms, reported 98% accuracy	98% accuracy	Limited dataset size
[41]	Combined CNN and KNN for potato plant disease recognition	CNN and KNN algorithms	90% accuracy	No details on the dataset and limitations
[42]	Data augmentation for potato plant leaves	Data augmentation techniques	Accuracy not reported	Focus on data augmentation, not classification accuracy
[43]	Compact CNNs for classifying tomato leaf diseases	CNNs with transfer learning	Higher accuracy	Not explicitly mentioned
[44]	Deep learning for tomato disease detection	Residual neural network	99.5% *F*1-score	Not explicitly mentioned
[45]	A comparative study on CNN variants for tomato plant disease recognition	CNNs	Higher classification accuracy	Computational expensive
[46]	Potato leaf disease detection	CNN variants	Higher accuracy	Not explicitly mentioned
[47]	Detection of sunn pest–damaged (SPD) wheat grains using deep learning	CNN variants	98.5% accuracy	Not explicitly mentioned
[48]	Classifying pepper seeds belonging to different cultivars using convolutional neural network (CNN) models	ResNet50	Higher accuracy	Not explicitly mentioned

**Table 2:** A table comparing the hyperparameters commonly used for ResNet-50 and ViT with multi-headed attention.

Hyperparameter	ResNet-50	Vision Transformer with Multi-Headed Attention
Input size	Variable	Fixed
Batch size	Variable	Variable
Learning rate	Typically 0.1	Typically 0.001
Optimizer	SGD, Adam, etc.	Adam
Weight decay	Typically 0.0001	Typically 0.1
Dropout rate	Typically 0.5	Typically 0.1
Number of layers	50	Variable (determined by model size)
Hidden size	N/A (Convolutional layers)	Variable (determined by model size)
Patch size	N/A (Convolutional layers)	Typically 16 × 16 or 32 × 32
Number of heads	N/A (Convolutional layers)	Variable (determined by model size)
Feed-forward size	N/A (Convolutional layers)	Variable (determined by model size)
Number of classes	Dependent on the dataset	Dependent on the dataset
Activation function	ReLU	GELU
Training schedule	Typically fixed number of epochs	Typically warm-up + linear decay
Image augmentations	Random cropping, flipping, etc.	Patch-wise augmentations

**Table 3:** List of classifications contained in the PlantVillage dataset that do not have any augmented data.

Potato Disease Class	Images	Training Set	Test Set
Class 1—Leaf Healthy	800	580	140
Class 2—Early Blight	1400	1060	260
Class 3—Late Blight	1400	1060	260
Total	3600	2700	660

**Table 4:** List of classifications in the PlantVillage dataset after data augmentation were applied [28].

Potato Disease Class	Images in Dataset	Training Set	Test Set
Class 1—Leaf Healthy	3600	2880	720
Class 2—Early Blight	3600	2880	720
Class 3—Late Blight	3600	2880	720
Total	10,800	8640	2160

**Table 5:** Training and validation loss on different deep learning models.

DL Methods	Train Loss	Train ACC (%)	Val Loss	Val ACC
TL1-ResNet-50	0.1765	91.56	0.2018	93.29
TL2-VGGNet-16	0.1935	93.14	0.2453	93.05
TL3-Xception	0.0210	93.64	0.0126	98.84
CNN-LSTM	0.1500	94.01	0.1752	96.25
TL4-VGGNet-16	0.1243	95.95	0.1568	93.75
T1-Swin	0.1335	95.98	0.1366	95.37
TL5-Inceptionv-3	0.0055	96.93	0.0136	98.09
Proposed Model	0.0201	99.87	0.0114	99.24

**Table 6:** Significance of the Proposed EfficientRMT-Net.

Model	Epochs	SE	SP	ACC	PR	*F*1-Score
EfficientNet	50	75	77	76	74	76.5
Vision Transformers (ViT)	50	80	82	84.1	83	82.6
depth-wise convolution (DWC)	50	82	84	84.6	83.5	84.5
ResNet	50	83	81	83.5	83	83.5
ViT + ResNet	50	82.5	82.5	80.1	83.5	84.6
EfficientNet + ResNet	50	81	84.2	84.3	84.5	85.5
ViT + DWC	50	86	87.5	86.5	85.5	85.0
EfficientRMT-Net (EfficientNet + ViT + DWC + ResNet)	50	94	96	97.6	94.12	96.2

SE: Sensitivity, SP: Specificity, RL: Recall, PR: Precision, ACC: Accuracy.

**Table 7:** State-of-the-art results of the proposed system model’s classification.

Model	Epochs	SE	SP	ACC	PR	*F*1-Score
Paul [23]—EfficientNet	50	78	80	79	76	79
Olawuyi [27]—Transfer	50	79	82	81.3	80	80.4
Nazir [28]—EfficientPNet	50	81	80	82.7	82	82.7
Chen [29]—Cnn	50	83	81	83.5	83	83.5
Barman [30]—DL	50	82	83	82.4	83	84.3
Mahum [31]—Efficient	50	84	84.2	84.3	84	85.2
Paul [23]—EfficientNet	50	85	86.2	87.6	85	86.1
Proposed Model	50	94	96	97.6	94.12	96.2

SE: Sensitivity, SP: Specificity, RL: Recall, PR: Precision, ACC: Accuracy.

**Table 8:** Performance evaluations of the proposed system model’s classification on Tomato and Grape datasets.

Benchmark	Epoch	Accuracy	*F*1-Score
Potato leaf	50	97.6%	96.2%
Tomato leaf	50	91.3%	90%
Grape leaf	50	90%	89.4%

## Data Availability

A standard online dataset, PlantVillage [32], is utilized in this paper to evaluate the EfficientRMT-Net model. It can be downloaded from https://data.mendeley.com/datasets/tywbtsjrjv/1 (accessed on 12 July 2023).
